# Perioperative mortality of emergency and elective surgical patients in a low-income country: a single institution experience

**DOI:** 10.1186/s13741-023-00341-z

**Published:** 2023-09-16

**Authors:** Samrawit Degu, Segni Kejela, Henok TekleSelassie Zeleke

**Affiliations:** 1 Department of Surgery, Lancet Biherawi General Hospital, Addis Ababa, Ethiopia; 2 Department of Surgery, College of Health Sciences, Addis Ababa University, Addis Ababa, Ethiopia

**Keywords:** Low-income countries, Perioperative mortality, Emergency surgery

## Abstract

**Background:**

The perioperative mortality rate is an indicator of access to safe anesthesia and surgery. Studies showed higher perioperative mortality rates among low- and middle-income countries. But the specific causes and factors contributing to perioperative death have not been adequately studied in the Ethiopian context.

**Methods:**

This is a retrospective institutional study of the largest academic medical center in Ethiopia. Data of all patients who were admitted to surgical wards or intensive care and underwent surgical interventions were evaluated for perioperative mortality rate determination. All mortality cases were then evaluated in depth.

**Results:**

Of the 3295 patients evaluated, a total of 148 patients (4.5%) died within 30 days of surgery. By the 7th postoperative day, 69.5% of the perioperative mortality had already occurred. Septic shock contributed to 54.2% of deaths. Emergency surgery patients had more than a twofold higher mortality rate than elective surgery patients (*p* value < 0.001) and had a 2.6-fold higher rate of dying within 7 days of surgery (*p* value of 0.02). Patients with ASA performance status of 3 or more had a 1.7-fold higher rate of death within 72 h of surgery (*p* value of 0.015).

**Conclusion:**

More than two thirds of patients died within 7 postoperative days. More emergency patients died than elective counterparts, and emergency cases had a higher rate of dying within 7 days of surgery. Poor ASA performance score was associated with earlier postoperative death. Further prospective multi-institutional studies are warranted to elucidate the factors that contribute to higher postoperative mortality in low-income country patients.

## Introduction

Perioperative mortality rate (POMR) is defined as the rate of death following surgery and anesthesia either on the day of surgery, before the 30th postoperative day or on the day of discharge from the hospital and is an indicator of access to safe anesthesia and surgery and (Aggarwal et al. 2020). POMR is inversely correlated with the improvement in access to advanced anesthesia care and surgical services (Bainbridge et al. 2012). This is why POMR is included in the World Health Organization’s 100 core health indicators list (Biccard et al. 2018).

Low-income countries (LIC) have a higher POMR than high-income countries (HIC) (Blaise Pascal et al. 2021; Bohnen et al. 2016). The Human Development Index (HDI) is also shown to associate with the rate of postoperative death when assessed on a global scale (Dandena et al. 2020). Few perioperative mortality studies have been reported from Ethiopia which consistently showed a high POMR (Davies et al. 2016; Dugani et al. 2017). As part of the Lancet Commission on Global Surgery’s core indicators for monitoring universal access to safe, affordable surgical and anesthesia care, it is planned to acquire a consistent reporting of the national POMR of all countries in the world by 2030 (Fecho et al. 2008). Accordingly, we are reporting a detailed assessment of POMR in the largest academic medical institution in one of the LIC, Ethiopia.

## Methods

### Study design

A retrospective cross-sectional study was conducted at a single referral hospital. All cases within the general surgery, pediatric surgery, neurosurgery, and cardiothoracic and vascular surgery units were analyzed. The study included all patients who were operated on and those who died within 30 days of surgery in the time course between May of 2021 and April of 2022.

### Study setting

The study was conducted at Tikur Anbessa Specialized Hospital, the largest teaching hospital in Ethiopia. It is located at the center of Addis Ababa, the capital city of the country. The hospital has over 700 beds and provides services to over 500,000 patients annually at a tertiary-level designation.

### Study participants

All surgical patients within the 4 surgical units selected for the study were included initially to determine the rate of POMR. Following this, perioperative mortalities (POMs) occurring during the course of the study were included and analyzed.

### Inclusion criteria

All surgical patients who underwent surgical intervention with open or minimally invasive techniques within the study period. All deaths following surgical intervention within 1 month after surgery regardless of the cause of death were included in the study and evaluated.

### Exclusion criteria

All patients with surgical disease who were treated non-surgically were excluded. In addition, all patients who died at the time of arrival at the hospital or before surgical intervention were excluded. Obstetric and gynecology, urology, and orthopedic patients were excluded. Obstetrics and gynecology cases were excluded because they are out of the jurisdiction of the Department of Surgery, and ethical clearance for these cases could not be acquired. Urology and orthopedics were excluded because there were no mortality cases during the course of the study. Finally, 17 cases with poor or incomplete documentation deemed difficult for analyses were also excluded.

### Variables

The independent variables for this study were gender, age, American Society of Anesthesiology (ASA) score, comorbidity, type of admission, indications for surgery, and surgical procedures performed.

The dependent variables were the rate of postoperative death, the cause of death, the postoperative day of death, and the length of hospital stay.

### Data source

The data source was from medical records, and both electronic and paper-based retrieved after the medical record numbers were acquired from the operation logbooks.

### Measurement/analysis and interpretation

After the data was collected, it was cleaned, coded, and entered into IBM Corp. Released 2015. IBM SPSS Statistics for Windows, version 23.0. Armonk, NY: IBM Corp. Both descriptive and inferential statistics were utilized for the interpretation of the data.

### Statistical analysis

For all categorical variables, measures of central tendency with mean and standard deviation were used in addition to frequency distribution. Inferential statistics with univariable and multivariable logistic regression was then performed for the risk identification regarding the time of death and associated variables.

### Ethical considerations

The ethical approval for this study was acquired from Addis Ababa University, College of Health Sciences, ethical review board. The study was conducted in accordance with the Helsinki Declarations, National and Institutional Guidelines, while keeping all the information retrieved for the study confidential.

## Results

**Fig. 1:**
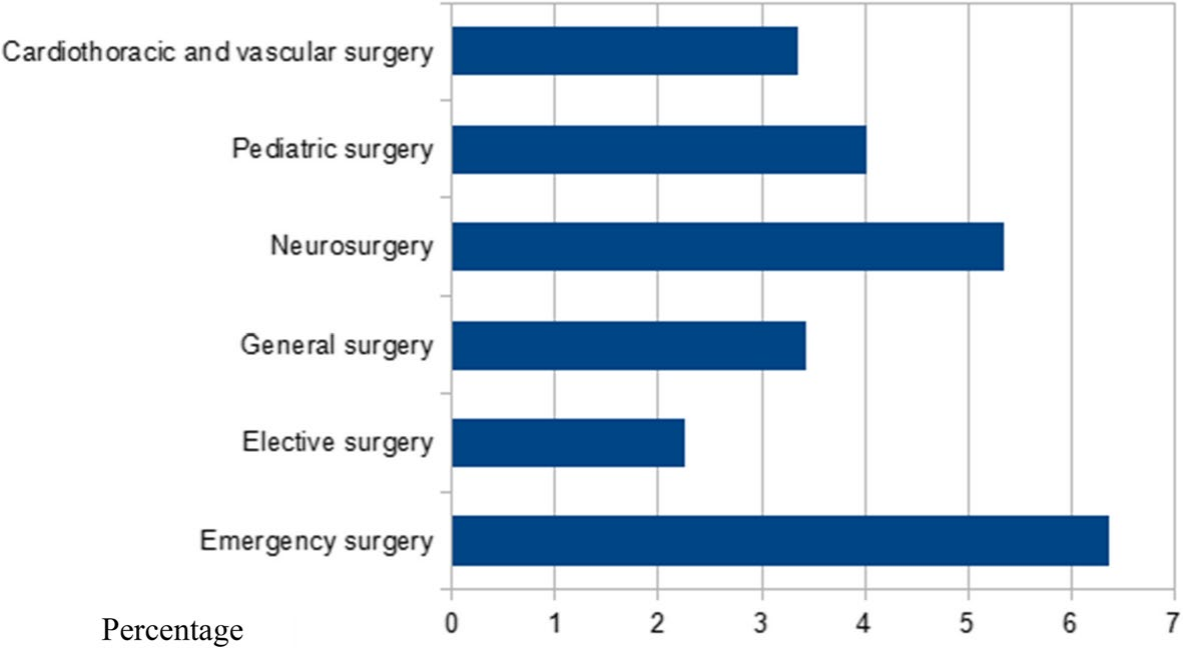
Percentage of death across different surgical units

Of the 3295 patients who underwent surgical interventions, 1246 belong to the pediatric age group. The total perioperative mortality was 148 (4.49%) of which 19 died within 24 h of surgery. The crude 24-h and 30-day perioperative mortality ratios were 1:173 and 1:22, respectively. Of the 148 deaths, 131 were analyzed while the rest were excluded due to incomplete documentation. A significantly higher rate of mortality was found in patients admitted for emergency surgery than elective surgery at 88/1384 (6.36%) and 43/1911 (2.25%), respectively, *p* value of < 0.001. Neurosurgery patients had higher overall POMR among the 4 units studied, which was 31/579 (5.35%), *p* value of 0.052 (Fig. 1 and Table 1).

**Table 1:** Death rate for each surgical sub-unit and admission type

Variables	Category	Number of deaths	Mortality rate(%)
Surgical units	General surgery	34/993	3.42
	Neurosurgery	31/579	5.35
	Pediatric surgery	50/1246	4.01
	Cardiothoracic and vascular surgery	16/477	3.35
Type of admission	Emergency surgery	88/1384	6.36
	Elective surgery	43/1911	2.25

**Fig. 2:**
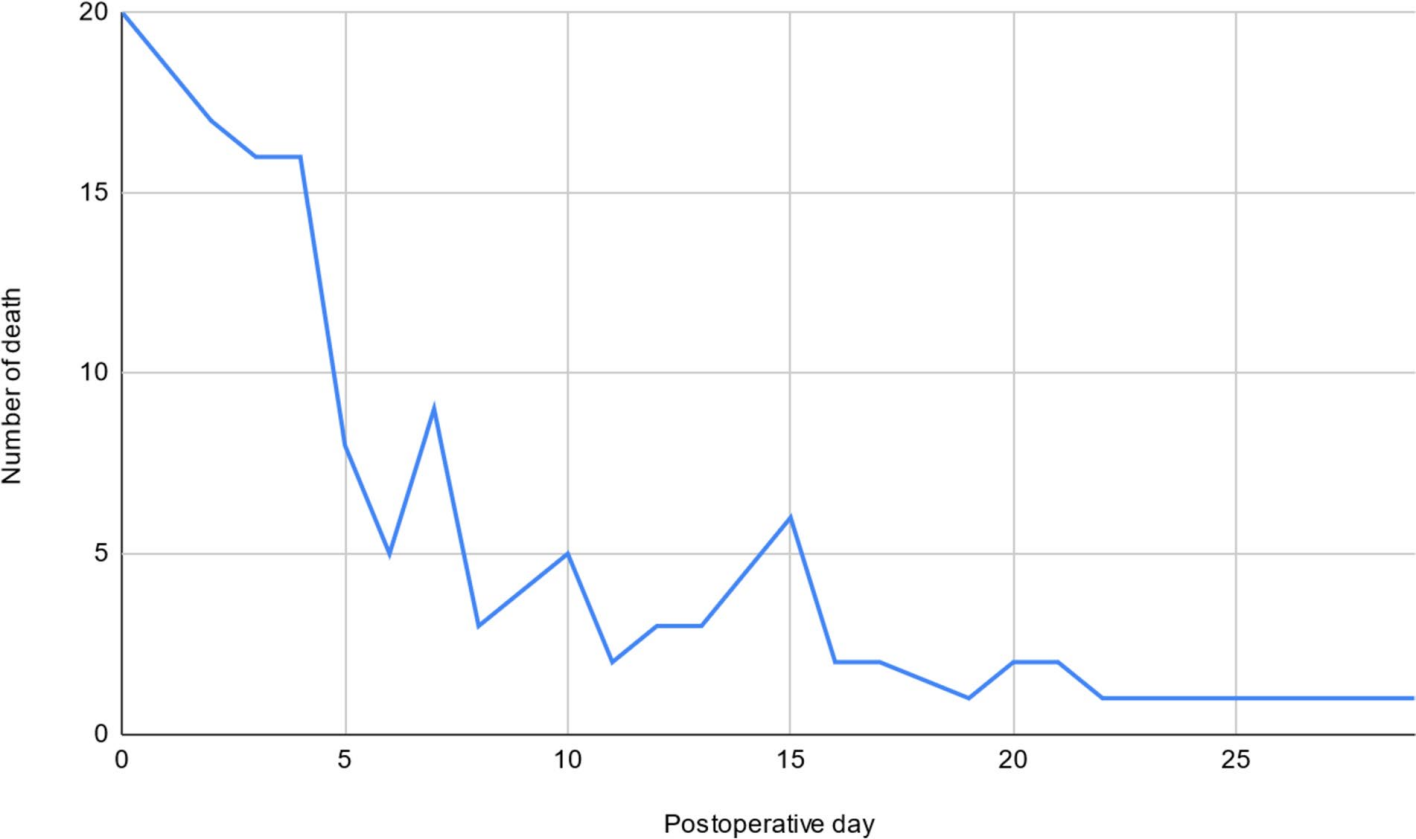
Trend of death across 1 month after surgery

From the 131 deaths evaluated, 69 (52.7%) were male. Patients in the neonatal age group were 41 (31.3%), and the mean age among the adult cases was 43.9 ± 18.5 years and 3.12 ± 2.59 days for infants. Overall, 53 (40.5%) died within 72 h of surgery, and 69.5% within 7 days of surgery (Fig. 2). The median overall postoperative day of death was 4 days. Patients older than 3 years of age had an earlier mean (5.28 days) and median (4 days) POM than their older counterparts (Table 2). This difference has shown a tendency towards statistical significance in one-way ANOVA, *F*(1129) = 3.77, *p* = 0.054.

**Table 2:** The mean and median of overall and postoperative stays of the emergency, elective cases, less than 4 years, and older age group

Category	Mean	Standard deviation	Range	Median
Overall POM day	6.89	6.68	0–29	4
Elective POM day	9.63	7.54	0–28	9
Emergency POM day	5.55	5.8	0–29	4
< 4 years POM day	4.8	4.77	0–21	4
≥ 4 years POM day	7.7	7.1	0–29	5
Overall hospital stay	13.46	10.2	1–53	10
Elective overall stay	20.7	9.9	4–53	19.5
Emergency overall stay	9.9	8.4	1–40	8

Elective and emergency surgical patients had a median POM day of 9 and 4 days, respectively, with a statistically significant difference in the average POM days between the two groups, *F*(1129) = 11.66, *p* = 0.001. The overall median length of hospital stay was 10 days (Table 2).

ASA score of 3 through 5 was recorded in 92 (70.2%) of the patients, and 24 (18.3%) had one or more medical comorbidity. During preoperative evaluation, anemia was detected in 82 (62.6%) of the mortality cases (Table 3).

**Table 3:** ASA scores, comorbidities, degree of anemia, and age group of the POMs

Variables	Category	Number of deaths	Mortality rate(%)
ASA score	ASA 1	6	4.6
	ASA 2	33	25.2
	ASA 3	54	41.2
	ASA 4	37	28.2
	ASA 5	1	0.8
Comorbidities‍	Hypertension	8	6.1
	Diabetes	4	3.1
	Hypertension + diabetes	2	1.5
	HIV	1	0.8
	Others	9	6.9
	None	107	81.7
Degree of anemia	None	49	37.4
	Mild (HGB 9.5–10.9 g/dl)	60	45.8
	Moderate (HGB 8–9.4 g/dl)	22	16.8
Age group	< 3 month	43	32.8
	3 month–14 years	6	4.6
	15 years–64 years	68	51.9
	65 years and above	14	10.7

**Fig. 3:**
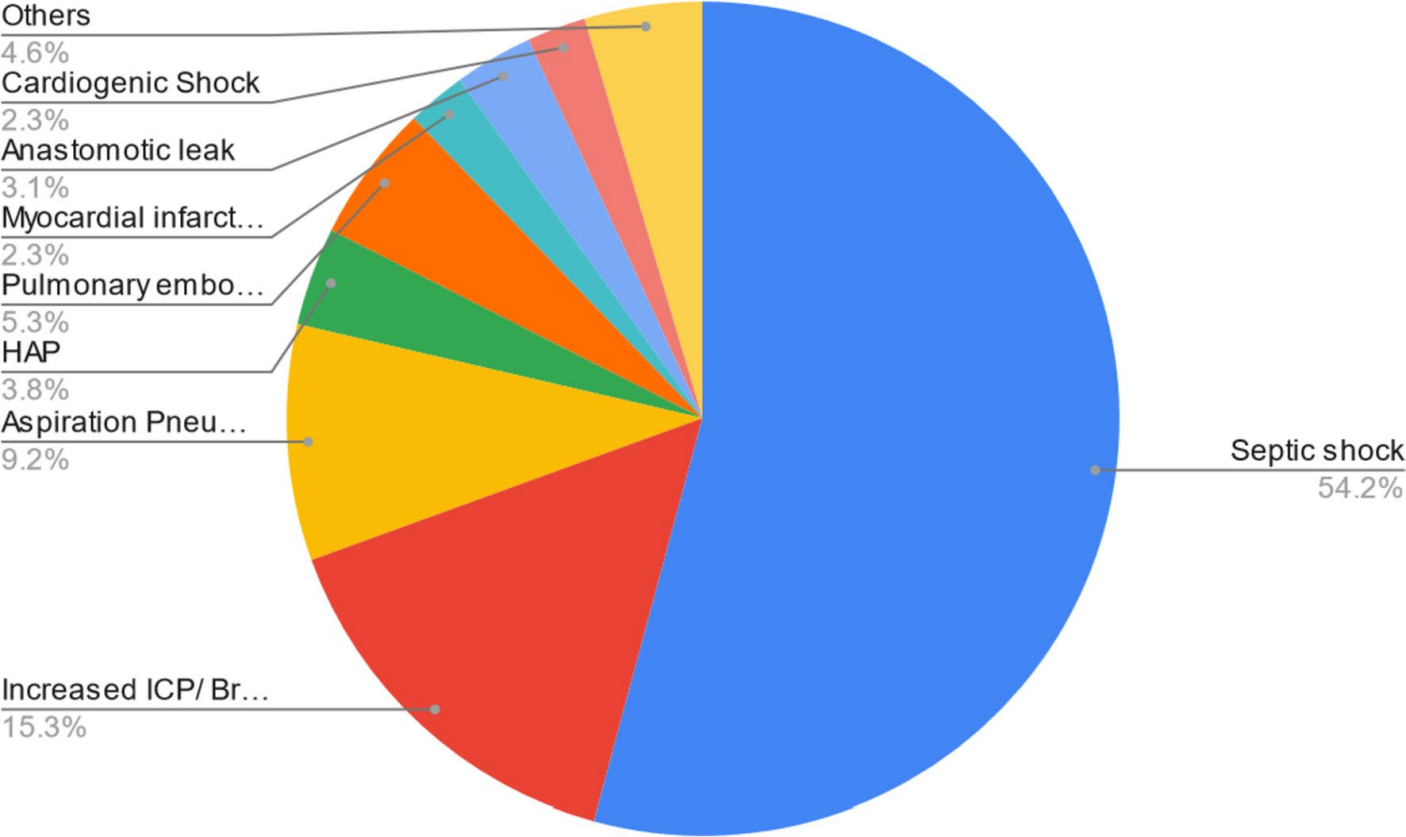
Cause of death of the cases. HAP hospital-acquired pneumonia

Regarding the indications for surgery, general surgical procedures were mostly done for generalized peritonitis due to viscus perforation and malignant intestinal obstructions. Elective surgery for brain tumors was the indication for surgery in 21 (67.7%) of deaths in neurosurgery. Overall, most deaths occurred due to septic shock (Fig. 3). The most common causes of death in neurosurgical patients were brain herniation and septic shock at 56.2% and 28.13%, respectively. Septic shock (63.3%) and aspiration pneumonia (20.4%) were the most common causes of death among the pediatric age group (Tables 3 and 4).

**Table 4:** Reasons for surgical admissions and immediate cause of death of the perioperative mortality cases among different surgical sub-units

Specialties	Reason for admission	Number of deaths	Percentage of deaths contributed (%)	Immediate cause of death	Number of deaths	Percentage of deaths contributed (%)
General surgery	Intestinal obstruction for malignancy	9	26.5	Septic shock	20	60.6
	Perforated viscus-benign	12	35.3	Myocardial infarction/pulmonary embolism	7	21.2
	Intestinal obstruction for benign cause	3	8.8	Aspiration pneumonia	2	6.1
	Perforated viscus-malignant	3	8.8	Hospital-acquired pneumonia	1	3
	Penetrating abdominal injury	2	5.9	Others	3	9.1
	Others	5	14.7			
Neurosurgery	Brain tumor	21	67.7	Brain herniation	18	56.3
	Traumatic brain injury	10	32.3	Septic shock	9	28.1
				Hospital acquired pneumonia	2	6.3
				Myocardial infarction/pulmonary embolism	1	3.1
				Others	2	6.3
Pediatric surgery	Tracheoesophageal fistula with/without EA/ARM	20	40.0	Septic shock	31	63.3
	ARM	7	14.0	Aspiration pneumonia	10	20.4
	Gastroschisis	6	12.0	Cardiogenic shock	2	4.1
	Intestinal atresia	5	10.0	Hospital-acquired pneumonia	1	2
	Midgut volvulus with rotation	3	6.0	Others	5	10.2
	Gangrenous intussusception	3	6.0			
	Others	6	12.0			
CTV surgery	Esophageal cancer	3	18.5	Septic shock	11	64.7
	Esophageal benign stricture	2	12.5	Myocardial infarction/pulmonary embolism	2	11.8
	Infectious lung conditions	2	12.5	Cardiogenic shock	2	11.8
	Gangrene 2° to PAD	4	25.0	Hospital-acquired pneumonia	1	5.9
	Vascular injuries	3	18.5	Others	1	5.9
	Others	2	12.5			

*EA* Esophageal atresia, *ARM* Anorectal malformation

Of the 19 POMs on the day of surgery, 18 (94.7%) died in the ICU, while one died in the wards. The majority, 56.5%, of the POMs beyond 24 h after surgery occurred in the ICU. No intraoperative death was recorded during the course of the study.

Multivariate logistic regression to evaluate factors that were associated with death within 72 h of surgery showed that only an ASA score of 3 or more was associated with a 1.7-fold increase in early postoperative death (AOR 1.73 (1.12–2.68), *p* value of 0.015). All the other factors analyzed including age group (infant versus older age group), admission circumstance (elective versus emergency), comorbidity, and gender did not affect the time of death (*p* value: 0.71, 0.13, 0.41, and 0.35, respectively). At postoperative day 7, only emergency surgery was associated with a 2.6-fold death rate in the first 7 days compared to the elective surgery group (AOR 2.65 (1.17–6.0) *p* value of 0.02). All other values including ASA score were found to be significantly associated with POMR at 7 days post-surgery.

## Discussions

In this study, the crude 24-h and 30-day perioperative mortality ratio was 1:173 and 1:22, respectively. Emergency surgery cases had close to a 2.5-fold higher mortality rate compared to their elective surgery counterparts. When comparing surgical services, higher POMR was recorded among neurosurgery patients, most of whom died from brain herniation. Patients who died within 24 h of surgery were in the ICU in nearly all of the cases, compared with only two thirds of those who died beyond 24 h. Septic shock was the most common cause of postoperative death. Patients with higher ASA scores (3 or more) had a 1.7-fold higher likelihood of dying within 72 h and emergency surgery deaths had a 2.6-fold likelihood of dying within the first 7 days than later. This finding reaffirms the existing body of evidence that early perioperative deaths are largely due to the inherently poor patient functional status and the emergent nature of the patient’s pathology.

A 30-day POMR of 2% was reported by Medecins Sans Frontieres across 3 nations within Central and Eastern Africa in a patient cohort comprising of mainly surgical emergencies of which the most common procedure was cesarean Sect.  (Fecho et al. 2008). A systematic review of studies from low- and middle-income countries showed an aggregate mortality rate of 1.2% for elective surgery and 10.1% for emergency surgery (Bainbridge et al. 2012). Furthermore, Ethiopian studies have shown a POMR rate ranging between 3.4% and 4.6% (Davies et al. 2016; Dugani et al. 2017). Our findings concur with both the national and regional findings, in that the POMR is higher than that of HIC, but equivalent to the regional figures (Fichtner and Dick 1997). In LIC, 40% of the POMR is reported to be attributed to facility-based resource factors such as postoperative care infrastructure and cancer care pathway, while the rest is attributed to patient factors (GlobalSurg Collaborative and National Institute for Health Research Global Health Research Unit on Global Surgery 2021). Consequently, the disparity between the two regions could partly be explained by the lack of infrastructure and human resources.

With regard to the immediate cause of death, a German multi-institutional study demonstrated that myocardial infarction and multiorgan failure were the primary causes of perioperative death (Hopkins et al. 2016). A Brazilian study showed that advanced disease and surgical complications were the most common causes of death among perioperative patients (Meara et al. 2016). One multinational LIC study on a 7-day cohort of perioperative outcomes put forward cardiovascular complications as the leading cause of death (Misganaw et al. 2022). Regardless, septic shock was the most predominant cause of death in the surgical population. In one LIC report, sepsis-related mortality is two-fold higher than that of the HIC patient population (Mullen et al. 2017). It could be hypothesized that mortality from sepsis could at least be partly attributed to the inadequate care provided at the LIC center and is likely to continue being an important cause of death in an LIC setting.

Aspiration pneumonia was found to be common among tracheoesophageal fistula/esophageal atresia patients. In one Ethiopian report, more than 90% of neonates with similar pathology had aspiration pneumonia. This is thought to emanate from neonates being fed prior to a late diagnosis leading to aspiration pneumonia (Ng-Kamstra et al. 2018).

Emergency patients in our study had higher POMR than their elective counterparts and a higher likelihood of death within the first 7 days of surgery. Our finding is in agreement with the existing body of knowledge that demonstrates that emergency and urgent surgery patients have 2–2.5 fold higher rate of mortality (Stefani et al. 2018; Tarekegn et al. 2020). To our knowledge, differences in the timing of POM between elective and emergency surgical patients have not been previously described, but a high rate of “early” postoperative death among emergency surgical patients has been reported (Watters et al. 2015). This could be explained by the inherent risk of the pathology that mandated the emergency surgery and the poor physiologic status of patients at presentation for acute care services (Weissman and Klein 2008).

The ASA performance status score was associated with “early” death among the cases studied here. Patients with an ASA score of 3 and above had a 2.9- to 16-fold increase in mortality within the first 72 h after emergency surgery (Weissman and Klein 2008). The association of ASA score with “early” POM has previously been shown among elective surgery patients, although the overall impact is relatively lower than the emergency surgical counterparts (World Health Organization 2015).

The future national plan needs to be made to create a registry in order to evaluate the aggregate multi-institutional POMR and factors associated with the POM including specific pathology, comorbidities, ASA scores, and postoperative complications, as these are not within the scope of this study. These registries need to be executed in accordance with the Lancet Global Surgery Initiative recommendations in order to produce a dataset of a comparable quality with the international literature.

In conclusion, we found a similar POMR rate in our study population as that in other low- and middle-income countries and higher than reports from HIC. Most deaths occurred due to septic shock and its complications. Emergency surgical patients had higher 7-day and 30-day mortality rates compared to their elective surgery counterparts. Additionally, patients with an ASA score greater than 2 had a two-fold increase in “early” death compared to those with better performance scores. These findings need to be confirmed using prospective multi-institutional, preferably national, studies.

## Data Availability

Source data will be available upon reasonable request to the corresponding author.
